# Definitions, terminology and standards for reporting of births and deaths in the perinatal period: International Classification of Diseases (ICD‐11)

**DOI:** 10.1002/ijgo.15794

**Published:** 2024-08-11

**Authors:** Hannah Blencowe, Lucia Hug, Ann‐Beth Moller, Danzhen You, Allisyn C. Moran

**Affiliations:** ^1^ Maternal, Adolescent, Reproductive and Child Health (MARCH) Center London School of Hygiene and Tropical Medicine London UK; ^2^ Division of Data, Analytics, Planning and Monitoring UNICEF New York New York USA; ^3^ UNDP‐UNFPA‐UNICEF‐WHO‐World Bank Special Program of Research, Development and Research Training in Human Reproduction (HRP), Department of Sexual and Reproductive Health and Research World Health Organization Geneva Switzerland; ^4^ Department of Maternal, Newborn, Child and Adolescent Health and Ageing World Health Organization Geneva Switzerland

**Keywords:** fetal death, ICD‐11, mortality statistics, neonatal death, perinatal, stillbirth

## Abstract

Despite efforts to reduce stillbirths and neonatal deaths, inconsistent definitions and reporting practices continue to hamper global progress. Existing data frequently being limited in terms of quality and comparability across countries. This paper addresses this critical issue by outlining the new International Classification of Disease (ICD‐11) recommendations for standardized recording and reporting of perinatal deaths to improve data accuracy and international comparison. Key advancements in ICD‐11 include using gestational age as the primary threshold to for reporting, clearer guidance on measurement and recording of gestational age, and reporting mortality rates by gestational age subgroups to enable country comparisons to include similar populations (e.g., all births from 154 days [22^+0^ weeks] or from 196 days [28^+0^ weeks]). Furthermore, the revised ICD‐11 guidance provides further clarification around the exclusion of terminations of pregnancy (induced abortions) from perinatal mortality statistics. Implementing standardized recording and reporting methods laid out in ICD‐11 will be crucial for accurate global data on stillbirths and perinatal deaths. Such high‐quality data would both allow appropriate regional and international comparisons to be made and serve as a resource to improve clinical practice and epidemiological and health surveillance, enabling focusing of limited programmatic and research funds towards ending preventable deaths and improving outcomes for every woman and every baby, everywhere.

## Synopsis

This paper summarizes new ICD‐11 guidance for standardized stillbirth and perinatal death reporting.

## 1 INTRODUCTION

Stillbirths are one of the world's most neglected tragedies. The estimated 1.9 million babies stillborn after 28 completed weeks of pregnancy in 2021 underestimates the overall burden of all stillbirths from 22 weeks.^1^ Each death has an important impact on affected women, families and healthcare providers.^2^

In 2014 the Every Newborn Action plan (ENAP), endorsed by all World Health Organization (WHO) Member States, included a stillbirth reduction target of 12 or fewer stillbirths in every country by 2030.^3^ United Nations Interagency Group for Child Mortality Estimation (UN IGME) now develops biannual national, regional and global stillbirth rate estimates,^1^ with regular WHO and United Nations Children's Fund (UNICEF) reports highlighting slower progress for stillbirth reduction, compared to neonatal and under‐five mortality.^4^, ^5^, ^6^ This increased attention has highlighted significant gaps in quality and comparability of stillbirth and perinatal death data globally.^7^

For more than a century, the International Classification of Diseases (ICD) has been the basis for comparable statistics on causes of mortality and morbidity between places and over time. The latest version of the ICD, ICD‐11, was adopted by the 72nd World Health Assembly in 2019 coming into effect on January 1, 2022.^8^

Despite standard ICD definitions for perinatal mortality in earlier ICD versions, challenges in understanding and practical application of these standard definitions, especially for the recording of fetal deaths and stillbirths persist. This results in substantial misclassification between stillbirths and neonatal deaths, and stillbirths and miscarriages, hindering data comparability between countries and over time.^9^, ^10^, ^11^

WHO, UNICEF, the WHO technical advisory group on maternal and newborn measurement (Mother and Newborn Information for Tracking Outcomes and Results [MoNITOR])^12^ and the Core Stillbirth Estimation Group (CSEG)^13^ of UN IGME collaborated to propose improved guidance for consistent stillbirth and perinatal death recording. The WHO ICD‐11 mortality review committee approved the proposal, and the update is reflected in the ICD‐11 reference guide from 2022.^8^

This paper summarizes new ICD‐11 guidance for standardized stillbirth and perinatal death reporting.

## 2 SUMMARY OF ICD‐11 GUIDANCE FOR REPORTING OF DEATHS IN THE PERINATAL PERIOD

### 2.1 Standards and reporting requirements for mortality in perinatal and neonatal periods

This ICD‐11 summary focuses on deaths in the perinatal and neonatal periods, including definitions to distinguish these from miscarriages. Where possible mortality systems should capture data on both fetal deaths and deaths following live birth in a manner that allows disaggregation between the two.

Accurate recording of events in the perinatal period (154 days [or 22 completed weeks, which is written as 22^+0^ to indicate 22 completed weeks and 0 days] of gestation to 7 completed days after birth) and neonatal period (0–27 days after birth) requires understanding of key terms used in the standard definitions to classify these events, including birth weight, gestational and chronological age (Table 1).

**TABLE 1:** Key terms used in perinatal definitions.

Key term	Definition
Gestational age	The duration of pregnancy estimated based on the best obstetric estimate of gestation. Gestational age is counted by calendar days where day 0 is used to refer to the first calendar day of gestation and day 1 for the second calendar day.^a^
Birth weight	The first weight of the fetus or neonate obtained after birth. For live births, birth weight should preferably be measured within the first hour of life before significant postnatal weight loss has occurred.
Chronological age^b^	The age since birth. Day 0 refers to the first 24 h after birth. Day 1 is the remainder of the second calendar day (date of death = date of birth^+1^) but outside the first 24 h. Day 2 is the third calendar day (date of death = date of birth^+2^).^c^
Fetal period	The period from 91 days of gestation (13^+0^ completed weeks) to birth.
Perinatal period	The period from 154 days of gestation (22^+0^ completed weeks) to 7 completed days after birth, i.e., includes days 0–6 after birth.
Neonatal period	The period from birth to 28 completed days after birth, i.e., includes days 0–27 after birth.

^a^ Record gestational age in calendar days to avoid confusion with interpretation of “completed weeks.” If needed, calculate completed weeks as (days since the first day of gestation)/7 and present as a whole integer plus a remainder in days (e.g., day 252 = 36^+0^/day 258 = 36^+6^).

^b^ Used in recording deaths in the neonatal period.

^c^ Where time of death is unknown: deaths where date of birth = date of death should be coded as day 0; and subsequently: Day of death = Date of death − date of birth.

### 2.2 Definitions used in perinatal and neonatal mortality

**FIGURE 1:**
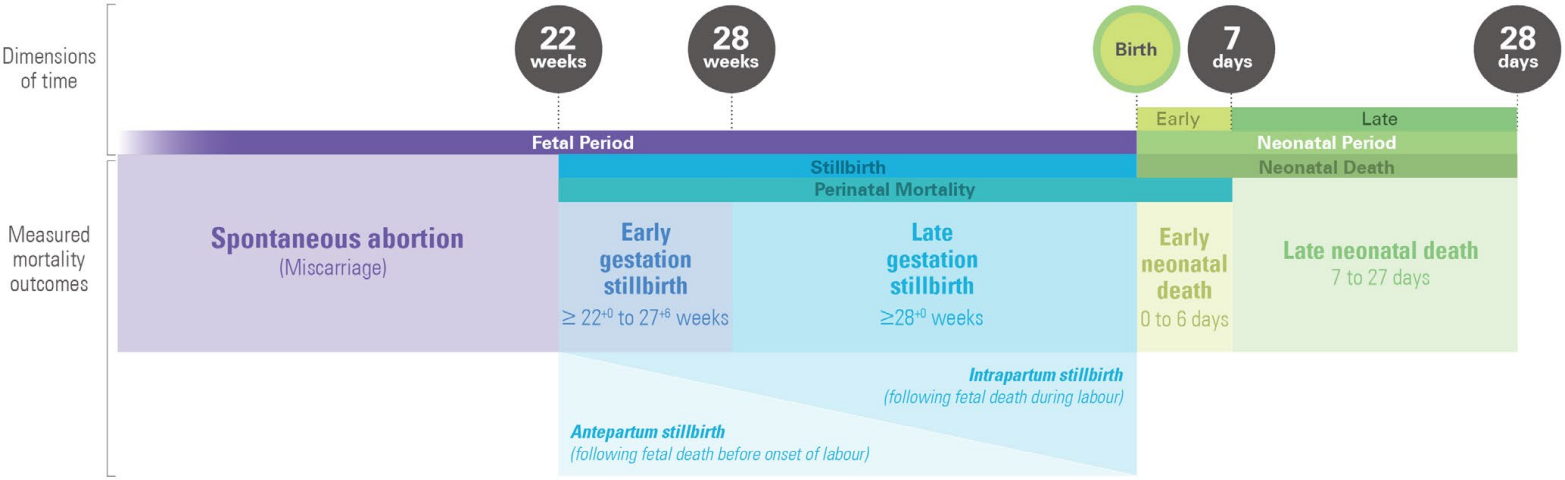
Mortality definitions related to the perinatal period.

Figure 1 gives an overview of these definitions.

#### Delivery

Deaths in the perinatal and neonatal periods are classified based on their timing in relation to delivery, defined as “the complete expulsion or extraction from a woman of a fetus.” In the definitions below we have used the more humane terms “birth” or “delivery” to refer to this process.

#### Fetal death

A fetal death is death of a fetus prior to birth, irrespective of the duration of pregnancy. Fetal death may be diagnosed in utero by absence of fetal heart sounds, confirmed by imaging techniques where available, or by the absence of signs of life after delivery or birth. Fetal deaths are categorized by their timing in relation to the onset of labor:

Antepartum fetal death is a fetal death before the onset of labor.
Intrapartum fetal death is a fetal death during labor.

#### Stillbirth

Whilst fetal death is the death of the fetus in utero, stillbirth refers to the delivery, or birth, of a baby after such a death. Delivery typically occurs spontaneously or planned in consultation with health providers and affected parents, within days of fetal death. Occasionally, weeks or longer between the fetus' death and its delivery or stillbirth.

A stillbirth is a baby born following a fetal death at 154 days (22^+0^ completed weeks) or more of gestation.

##### Gestational age subgroups

Early gestation stillbirth is a stillbirth between 154 and 195 days of gestation (22^+0^–27^+6^ weeks).

Late gestation stillbirth is a stillbirth at 196 or more days gestation (≥28^+0^ weeks).

If no gestational age information is available birth weight ≥500 g can be used as a proxy for stillbirth, with early gestation stillbirths ≥500 to <1000 g and late gestation stillbirths ≥1000 g.

##### Stillbirths by timing

Antepartum stillbirth is a stillbirth following antepartum fetal death (before onset of labor). Intrapartum stillbirth is a stillbirth following intrapartum fetal death (occurring during labor).

Every effort should be made to assess and record the vital status of the fetus for all women presenting in labor to the health facility. Skin appearance is a poor proxy for stillbirth timing and should only be used when the vital status of the baby at the onset of labor, or admission to the health facility is unknown.^11^, ^14^ In these cases macerated stillbirth (presence of maceration at delivery) suggests antepartum death, while fresh stillbirth (no maceration) suggests intrapartum death.

#### Live birth

A live birth is the delivery of a baby, irrespective of the duration of the pregnancy, which after such separation shows signs of life. Signs of life at birth include breathing, beating of the heart, pulsation of the umbilical cord and definite movement of voluntary muscles whether the umbilical cord has been cut or the placenta is attached. Fleeting reflex activity, defined as automatic involuntary reflexes triggered by stimuli such as touch or temperature changes, observed only in the first minute after birth does not warrant classification as a sign of life.

#### Total births

The sum of stillbirths and live births. If the lower limit of stillbirth used differs from 154 days (22^+0^ completed weeks), for example ≥196 days (≥28^+0^ weeks) for international reporting, this should be clearly stated.

#### Neonatal death

A neonatal death is a death during the first 28 completed days after live birth (days 0–27).

##### Groupings by chronological age

An early neonatal death is a death during the first 7 completed days after live birth (days 0–6).

A late neonatal death is a death 7–27 days after live birth.

### 2.3 Recommendations for recording of perinatal events

Reporting requirements for mortality statistics differ from individual‐level recording requirements to guide clinical care. To facilitate accurate reporting ICD‐11 recommends a minimum perinatal dataset be recorded for all births, including stillbirths, with gestational age and birth weight recorded to the degree of accuracy to which they measured (Table S1). Gestational age should ideally be recorded in days. If recorded in weeks, separate register columns for completed weeks and number of days since last completed week are recommended. For accurate birth weight reporting, record the actual weight measured (ideally using a calibrated electronic weighing scale to the nearest 10 g) instead of using pre‐defined weight categories (e.g., 500 g groupings) employed for statistical tabulation. Record chronological age at death in completed minutes or hours for the first 24 h (day 0), then days thereafter (days 1–27).

### 2.4 Recommendations for reporting of perinatal events

All births, both live and stillborn, and deaths can be categorized by period of gestation and birth weight for reporting purposes. Neonatal deaths can also be categorized by chronological age.

#### Categorization by period of gestation

**FIGURE 2:**
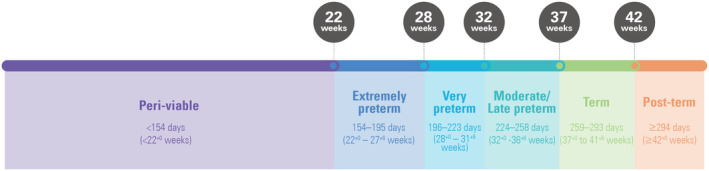
Classification of births by period of gestation. Where the peri‐viable category is used the lower gestational limit for inclusion should be specified.

Gestational age is used to define maturity: preterm (<37^+0^ weeks, <259 days), term (37^+0^ to 41^+6^ weeks, 259–293 days), and post‐term (≥42^+0^ weeks, ≥294 days). Preterm births can be further subgrouped as peri‐viable, extremely, very, moderate and late preterm (Figure 2).

#### Categorization by birth weight

**FIGURE 3:**
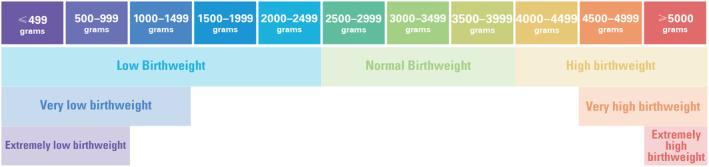
Classification of births by birth weight. Low birth weight (LBW) and high birth weight can be further categorized into “extremely” and “very” subcategories. As these terms are not mutually exclusive, reporting using the 500 g intervals as above is recommended.

For birth weight reporting of fetal deaths under 22 weeks, stillbirth and neonatal mortality statistics intervals of 500 g should be used, that is, ≤499; 500–999; 1000–1499; 1500–1999; 2000–2499; 2500–2999; 3000–3499; 3500–3999; 4000–4499; 4500–4999; and ≥5000 g (Figure 3).

#### Categorization by chronological age

Neonatal deaths can be further categorized by chronological timing since birth. Definitions of early and late neonatal deaths are given above. For mortality statistics, reporting by single days is recommended for the first week of life (days 0–6), and then by week (week 2 [7–13 days], week 3 [14–20 days], week 4 [21–27 days]). Additionally, where possible, grouping day 0 deaths into <1, 1–11 and 12–23 h is recommended.

### 2.5 Other considerations in the reporting of perinatal and neonatal deaths

#### Lower thresholds for reporting of deaths

ICD‐11 recommends including all stillbirths and deaths following live birth if gestational age is at least 154 days (≥22^+0^ weeks). When gestational age is unavailable, the birth weight proxy (≥500 g) can be used. If the gestational age and birth weight are unknown it is recommended to include the case in the perinatal mortality statistics if there is a strong probability the event occurred after the defined reporting point for perinatal mortality.

##### Fetal deaths

National reporting requirements for fetal deaths vary, with legal thresholds often closer to the boundaries of fetal viability for example, 20^+0^ to 28^+0^ weeks (140–196 days) than the start of the fetal period. Recording fetal deaths from 140 days (20^+0^ weeks) in local health data collection systems can improve capture and accuracy of reporting from 22^+0^ weeks. The lower limit used for data recording should be specified in the statistics produced.

##### Deaths following live birth

ICD‐11 recommends recording all deaths following live birth, regardless of gestational age. Disparities in capturing and recording neonatal deaths, especially at lower gestational ages, pose a challenge for international comparisons of mortality rates.^15^, ^16^, ^17^ To address this, ICD‐11 offers clear reporting guidelines to ensure consistency across countries (see below).

#### Recording of pregnancy losses occurring before the perinatal period

A spontaneous abortion (or miscarriage) is the spontaneous loss of pregnancy (i.e., embryo or fetus) prior to 154 days (22^+0^ weeks) gestation. To enable consistency in comparison across countries, spontaneous pregnancy losses <154 days should always be classified as miscarriages and those ≥154 days as stillbirths for international reporting, regardless of local reporting requirements.

#### Recording of other events in the perinatal period

The above guidance relates to stillbirth and neonatal mortality only and does not include induced abortions, a minority of which occur at or after 154 days (22^+0^ weeks).

Artificial termination of an ongoing pregnancy (also referred to as induced abortion, legal abortion, fetal reduction) is defined by ICD‐11 as the complete expulsion or extraction from a woman of an embryo or a fetus (irrespective of the duration of the pregnancy), following a deliberate interruption of an ongoing pregnancy by medical or surgical means, which is not intended to result in a live birth.

All events meeting the definition of artificial termination of pregnancy should be reported in mortality statistics but must be clearly distinguishable from spontaneous abortions and stillbirths. Whilst transient signs of life may be present in later‐gestation termination, these should never be coded as live births and neonatal deaths.

### 2.6 Death certification

Accurate child mortality data (including stillbirths) requires complete recording, reporting, and classification of births and deaths. ICD‐11 recommends cause of death certificates for all perinatal deaths, fetal or neonatal, using the standardized international form which has a dedicated “Fetal or Infant Death” section (Table S2).

Every effort should be made by death certifiers to differentiate between stillbirth (following a fetal death) and neonatal death (following a live birth), using information from the medical records, labor ward registers, perinatal audit and other sources.

### 2.7 Reporting criteria for international comparisons

Inclusion of fetal deaths and live births born at extremely low gestational ages disrupts the validity of international comparisons and is therefore not recommended.

For international comparisons countries should report all late gestation stillbirths (≥196 days [≥28^+0^ weeks] or birth weight ≥1000 g, if gestational age unknown). Where possible, additionally report early gestation stillbirths (154–195 days [22^+0^–27^+6^ weeks] or birth weight 500–999 g, if gestational age unknown) as a separate category (Table 2).

**TABLE 2:** Recommendations for internationally comparable reporting of perinatal death statistics.

Gestational age at delivery/birth in completed days (weeks)	Birth weight equivalent (only if gestational age not available (g)	Vital status at delivery/birth		Notes
		Non‐livebirth	Livebirth followed by neonatal death	
<154 days (<22^+0^ weeks)	<500	Miscarriage^a^	Very early gestation neonatal death	Only required for neonatal mortality national statistics. When used for spontaneous abortion (miscarriages), should be reported separately from perinatal statistics and the lower gestational age limit for data collection stated
154–<196 days (22^+0^–27^+6^ weeks)	500–999	Early gestation stillbirth	Early gestation neonatal death	For national statistics, and for international statistics of countries with ability for reporting of early gestation deaths (stillbirths and neonatal mortality)
≥196 days (≥28^+0^ weeks)	≥1000	Late gestation stillbirth	Late gestation neonatal death	For international statistics (stillbirths and neonatal mortality)
Unknown gestational age	Unknown birth weight	Stillbirth (unknown gestation)	Neonatal death (unknown gestation)	Include in statistics only when there is a high likelihood that the stillbirth or neonatal death occurred at the given criteria, e.g., 28 or more weeks for international statistics

*Note*: This table excludes events resulting from artificial termination of an ongoing pregnancy (induced abortions).

^a^ At very early gestations spontaneous onset of preterm birth may result in a miscarriage without a preceding fetal death.

Whilst all neonatal deaths should be reported in the national data system, for international comparisons only those born ≥196 days (≥28^+0^ weeks or birth weight ≥1000 g, if gestational age unknown) should be included. This recommendation represents a shift from previous recommendations and current practice in many countries. However, gestational age and birth weight should now be recorded for every birth and death, and national reporting systems should be adapted to facilitate reporting using these thresholds.

Where deaths before 154 days (<22^+0^ weeks or <500 g, if gestational age unknown) are included in perinatal statistics they should be presented separately from deaths at 154 days or more (≥22^+0^ weeks or <500 g, if gestational age unknown) and the lower limit for inclusion in perinatal statistics in the setting should be stated, for example, “20^+0^ weeks of gestation” or “no lower gestational age limit.”

#### Indicators for international comparisons of perinatal mortality

To facilitate international comparisons, ICD‐11 recommends reporting the following rates:

Late gestation stillbirth rate = ([stillbirths ≥28^+0^ weeks]/[total births ≥28^+0^ weeks]) × 1000.
Early neonatal mortality rate = ([day 0–6 neonatal deaths born at ≥28^+0^ weeks]/[live births born at ≥28^+0^ weeks]) × 1000.
Perinatal mortality rate = ([stillbirths ≥28^+0^ weeks + day 0–6 neonatal deaths born at ≥28^+0^ weeks] /[total births ≥28^+0^ weeks]) × 1000.

## 3 DISCUSSION

The WHO, through the ICD, has been producing guidance for the reporting of perinatal deaths for over 70 years.^18^ Despite efforts to provide clarity on definitions for reporting of perinatal mortality, including stillbirths and neonatal death, there remains substantial heterogeneity in the understanding and application of standard definitions, limiting comparability of these data between countries and over time. This in part has resulted from the large changes in the field of maternal and perinatal health. These decades have seen large advances in clinical obstetric and neonatal care, with the advent of interventional obstetrics and neonatal intensive care changing perceptions of viability, and in measurement, with the increasing recognition of the importance and ability to measure, gestational age.

Since ICD‐10 was introduced in 1990, there have been large increases in understanding of epidemiology and measurement of perinatal mortality. Work using routine data from high‐income settings led through the EURO‐PERISTAT project, North American and Australian researchers have highlighted some of the challenges in comparability between these settings.^15^, ^16^, ^17^, ^19^, ^20^ Additionally, work has been undertaken to improve understanding of these data from a global perspective, seeking to better understand perinatal mortality measurement in data systems commonly used in high burden settings, such has household surveys.^9^, ^11^, ^21^, ^22^, ^23^, ^24^, ^25^ For this revision (ICD‐11), we brought together perinatal epidemiologists and measurement experts with experience across a wide range of contexts as part of the Mother and Newborn Information for Tracking Outcomes and Results (MoNITOR) and the Core Stillbirth Estimation Group (CSEG) of the UN IGME to produce a unified proposal to align and simplify the recommended standard definitions. Key learnings from these researchers included in the revised ICD‐11 guidance include: using gestational age as the primary threshold to be used for reporting, providing further guidance on the measurement and recording of gestational age, reporting mortality rates by gestational age subgroups to enable country comparisons to include similar populations, for example, all births from 154 days (22^+0^ weeks) or from 196 days (28^+0^ weeks) and clearer guidance around exclusion of terminations of pregnancy (induced abortions) from perinatal mortality statistics. The guidance also recommends that gestational age be recorded in calendar days to minimize confusion often associated with the interpretation of completed weeks with additional days. In addition, this version of ICD standardized spelling of key terms, for example, fetal, stillbirth and live birth across the guidance.

### 3.1 Improving the measurement of stillbirths and neonatal deaths

There is now increasingly widespread agreement that most stillbirths and neonatal deaths globally are preventable. With 194 government signatories to the Every Newborn commitment to reduce stillbirths to 12 or fewer per 1000 total births and neonatal deaths to 12 or fewer per 1000 livebirths by 2030 there has also been increasing investment and political traction towards meeting these targets.^3^, ^6^ However, despite this, large gaps exist in national‐ and global‐level data on stillbirths and neonatal deaths.^5^, ^7^ Urgent investments are needed to close these data gaps in order to end these unnecessary deaths by 2030.

Under‐reporting and misclassification are common, especially for stillbirths and early neonatal deaths. Reducing omission will require strengthening of data systems to capture these deaths, including identifying and addressing barriers to capturing every birth and death, including blame and fear of punitive action.^26^ Improving the measurement of stillbirths and neonatal deaths and reducing misclassification will necessitate efforts to improve the measurement of key components required to accurately classify these deaths—including birth weight, gestational age and vital status at birth. Important steps are being taken towards strengthening accurate routine measurement of gestational age, including WHO recommendations for at least one ultrasound during antenatal care.^27^ Advancements in Artificial Intelligence hold promise for significantly improving the accuracy of ultrasound‐based gestational age assessment, particularly in resource‐limited settings where access to trained sonographers might be limited.^28^ This technology could also prove beneficial for ultrasounds performed later in pregnancy, when accurate dating can be more challenging.

Improving available data will require a multifaceted approach including improving healthcare providers' skills and competencies for reporting. The use of the WHO minimum perinatal dataset at the health facility level is an important first step (Table S1).^29^ Further steps which could be considered include recording gestational age in calendar days to minimize confusion often associated with the interpretation of completed weeks with additional days. UNICEF and CSEG have recently produced guidance on “Stillbirth Definition and Data Quality Assessment for Health Management Information Systems (HMIS).”^30^ This document aligns with ICD‐11 and provides practical guidance on how the recording of stillbirth data can be strengthened in routine healthcare settings.

### 3.2 Improving comparable reporting of deaths in the perinatal period

ICD‐11 provides guidance for the minimum data required to enable between country comparison. Individual countries, or subnational regions, may have additional reporting priorities and require a wider range of data to be collected. However, to enable valid international comparisons ICD‐11 recommends that local and national registration and reporting procedures should be arranged to ensure that the events and the criteria for their inclusion in the statistics for international comparison can be easily identified and extracted for global reporting.

### 3.3 Cause of death reporting

The present review has focused on the reporting of the death event and timing of the death, not the cause of death. Reporting of perinatal deaths by timing is an important marker of quality of care.^11^, ^31^, ^32^ At a minimum it requires assessment of fetal heart rate on admission in labor and vital status at birth, both of which should be routinely assessed for births in a facility by the healthcare provider. However, whilst these are usually assessed by health workers, although vital status at birth is recorded in routine data systems few low‐ and middle‐income settings record fetal heart rate on admission. Further efforts are needed to capture these data in health records and registers and provision made for these data to be collated for reporting at a local, regional and national level. Improving its measurement should be the first step towards enhancing data for deaths in the perinatal period, investments will also be needed in improving data around cause of death. Whilst accurate reporting of levels and timings of deaths in the perinatal period give high‐level insights into potential areas to target health systems investments, for example, if very high levels of intrapartum stillbirths are observed investment in referral and emergency obstetric and neonatal care systems could be targeted. However, to inform investments into targeted programmatic action and to close remaining implementation and biomedical research gaps will require investment to further understanding of the individual causes of death.

ICD‐11 also covers the standardized reporting of the causes of death in the perinatal period. In 2016 for the previous version of ICD, ICD‐10, WHO produced guidance for a standardized system for classifying causes of stillbirth and neonatal deaths globally: “The WHO application of ICD‐10 to deaths during the perinatal period: ICD‐PM.”^33^ This is an important step towards comparable estimates of cause of deaths in the perinatal period, especially for stillbirths where large number of classification systems current in use have limited comparability.^34^, ^35^, ^36^ However, a recent review found that ICD‐PM is applied inconsistently and suggesting possible solutions such as including a category for perinatal deaths of unknown timing, providing clearer explanations for consistent coding, and highlighting potential pitfalls of the system.^37^ Updated global guidance on classifying perinatal deaths according to the new ICD‐11 guidance, incorporating experiences and learnings from users of the ICD‐10 version, would be an important next step towards comparable cause of death estimates for this period.

## 4 CONCLUSION

High quality comparable perinatal mortality data are critical to track progress towards global goals to end preventable deaths for stillbirth and newborn. Despite recent large increases in the proportion of all births taking place in health facilities where labor ward records are ubiquitous, large gaps currently exist in the availability and quality of data particularly in low‐ and middle‐income countries. Implementation of the ICD‐11 standard methods for recording and reporting all births and deaths in the perinatal period in every setting would be an important first step towards improving data. High quality data would both allow appropriate regional and international comparisons to be made and serve as a resource to improve clinical practice and epidemiological and health surveillance, enabling focusing of limited programmatic and research funds towards improving outcomes for every woman and every baby, everywhere.

## AUTHOR CONTRIBUTIONS

All authors were involved in the design and planning of this work. Hannah Blencowe wrote the first draft of the manuscript, all authors reviewed and contributed to revising the manuscript. All authors approved the final version.

## FUNDING INFORMATION

Bill & Melinda Gates Foundation.

## CONFLICT OF INTEREST STATEMENT

The authors confirm there are no conflicts of interest.

## AUTHOR DISCLAIMER

The authors alone are responsible for the views expressed in this article and they do not necessarily represent the views, decisions, or policies of the institutions with which they are affiliated.

## Supporting information


Figure S1.

Table S1.
Table S2.

## Data Availability

Data sharing is not applicable to this article as no new data were created or analyzed in this study.
