# Supplementary figures and images for: Definitions, terminology and standards for reporting of births and deaths in the perinatal period: International Classification of Diseases (ICD‐11)
Source: Int J Gynaecol Obstet. 2024 Aug 11;168(1):1–9. doi: 10.1002/ijgo.15794 (PMC11649847; doi:10.1002/ijgo.15794)

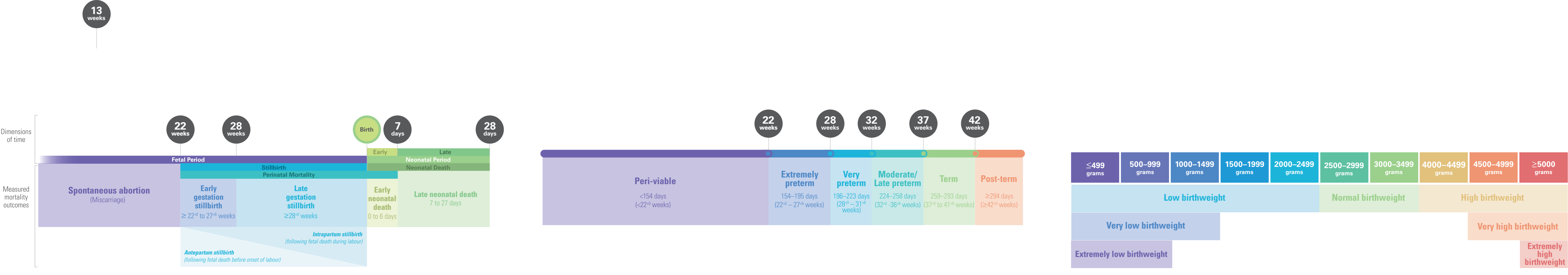

Supplement: Supplementary file 1 — Figure S1. [file IJGO-168-1-s002.pdf]
