# Supplementary material for: Definitions, terminology and standards for reporting of births and deaths in the perinatal period: International Classification of Diseases (ICD‐11)
Source: Int J Gynaecol Obstet. 2024 Aug 11;168(1):1–9. doi: 10.1002/ijgo.15794 (PMC11649847; doi:10.1002/ijgo.15794)

Table S1: Minimum perinatal dataset (WHO)

| **All births:** |
| --- |
| ID # mother: |
| ID # baby: |
| Facility name: |
| District name: |
| Obstetric history: Number of pregnancies, number of live births |
| Mother’s age (years): |
| Type of pregnancy: singleton, twin, higher multiple |
| Number of antenatal care visits: |
| HIV-status: |
| Mother’s last menstrual period: |
| Date of birth – time of birth: |
| Gestational age (in days or completed weeks plus remaining days) – method of determination: |
| Place of delivery: |
| Birth attendant: midwife, nurse, doctor, other, unknown |
| Mode of delivery: cephalic vaginal, breech vaginal, caesarean section |
| Sex of baby: |
| Birthweight (grams): |
| **If deceased, the following additional information should also be collected:** |
| Date of death – time of death: |
| Type of death: neonatal, intrapartum stillbirth, antepartum stillbirth, stillbirth unknown timing |

*Note:* World Health Organization. Making every baby count. Audit and review of stillbirths and neonatal deaths. https://www.who.int/publications/i/item/9789241511223 2016.

Table S2: The International Form of Medical Certificate of Cause of Death


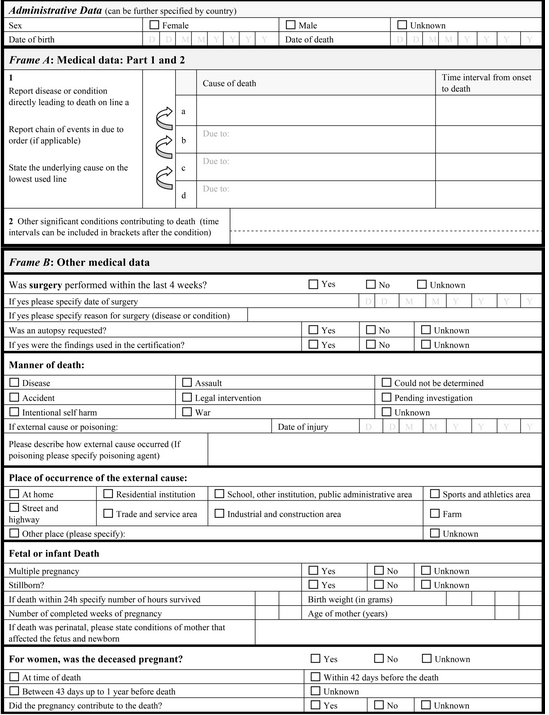

Supplement: Supplementary file 2 — Table S1. Table S2. [file IJGO-168-1-s001.docx]
